# Impact of seborrheic dermatitis on osteoporosis risk: A population‐based cohort study

**DOI:** 10.1111/1346-8138.16578

**Published:** 2022-09-12

**Authors:** Ying‐Yi Lu, Chun‐Ching Lu, Cheng‐Yu Tsai, Yao‐Ju Liu, Chao‐Lan Huang, Wei‐Ting Wang, Chieh‐Hsin Wu

**Affiliations:** ^1^ Department of Dermatology Kaohsiung Veterans General Hospital Kaohsiung Taiwan; ^2^ Department of Nursing Shu‐Zen Junior College of Medicine and Management Kaohsiung Taiwan; ^3^ Department of Post‐Baccalaureate Medicine, School of Medicine, College of Medicine National Sun Yat‐sen University Kaohsiung Taiwan; ^4^ Department of Orthopaedics and Traumatology National Yang Ming Chiao Tung University Hospital Yilan Taiwan; ^5^ Department of Orthopaedics, School of Medicine National Yang Ming Chiao Tung University Taipei Taiwan; ^6^ Department of Orthopaedics and Traumatology Taipei Veterans General Hospital Taipei Taiwan; ^7^ Division of Neurosurgery, Department of Surgery Kaohsiung Medical University Hospital Kaohsiung Taiwan; ^8^ Department of Surgery, School of Medicine, College of Medicine Kaohsiung Medical University Kaohsiung Taiwan; ^9^ Department of Anesthesiology Taipei Veterans General Hospital Taipei Taiwan; ^10^ Department of Anesthesiology, School of Medicine National Yang Ming Chiao Tung University Taipei Taiwan; ^11^ Department of Radiology, Tri‐Service General Hospital National Defense Medical Center Taipei Taiwan; ^12^ Center for Big Data Research Kaohsiung Medical University Kaohsiung Taiwan

**Keywords:** bone health, inflammation, osteoporosis, population, seborrheic dermatitis

## Abstract

Osteoporosis is a systemic bone‐resorbing disease that easily causes subsequent risk of fracture. Hence, the substantial physical burden of osteoporosis makes it an important public health issue. Seborrheic dermatitis (SD) is a chronic, recurrent, inflammatory skin disease. Despite the advances in medication for treating osteoporosis, identifying undiagnosed osteoporosis patients is still challenging. Since osteoporosis and SD share a similar pathobiology, e.g. inflammation and hormonal imbalance, we aimed to investigate whether the existence of SD increases osteoporosis risk by using the Taiwan National Health Insurance Research Database. A total of 7831 patients aged 18–50 years with SD and a control group of 31 324 patients without SD matched by age, gender, Charlson Comorbidity Index, and index date at a ratio of 1:4 during 1996–2010 were recruited in the study. To measure the cumulative incidence and compare the hazard ratios of osteoporosis between each group, the Kaplan–Meier method and Cox proportional hazard regression models were utilized. It was found that 0.98% of SD patients had osteoporosis. Compared to the non‐SD group, the SD group had a 5.95‐fold higher osteoporosis risk after adjustment for variables. The impact of SD on osteoporosis risk was largest in the female and young age groups. In addition, the presence of hyperlipidemia, hyperthyroidism, and epilepsy synergistically increased osteoporosis incidence in the SD group. This first large cohort study demonstrated an association between SD and osteoporosis. Since the effect on bone health in SD patients with concomitant diseases is largest in early life, diet or lifestyle recommendations as well as regular bone examinations are advised during follow‐up of SD.

## 1 INTRODUCTION

The substantial physical burden of osteoporosis makes its growing incidence in the general population an important public health issue.^1^ Osteoporosis is a systemic bone‐resorbing disease that causes bone fragility and subsequent risk of fracture.^2^ Globally, osteoporosis is the most prevalent bone disorder.^3^ The disease is more common in females than in males,^4^ and osteoporotic fracture occurs in more than half of all females older than 50 years.^5^ The disease results from an osteoclast–osteoblast imbalance caused by hormonal deficiency and abnormal increase in systemic pro‐inflammatory cytokines.^6^ Despite recent remarkable advances in medications for treating osteoporosis,^7^ identifying undiagnosed osteoporosis patients is still challenging.

Seborrheic dermatitis (SD) is an inflammatory skin disease. Clinical manifestations include ill‐defined erythematous exfoliative scaling patches accompanied by variable pruritus. The disease is often chronic, and relapse is common.^8^ The most common sites of SD, known as “seborrheic areas,” are the anterior chest, axilla, back, groin, central area of the face, and scalp.^9^, ^10^ Its incidence is highest in infants, adolescents, and adults aged 30–60 years.^11^ The prevalence of SD is approximately 5% in adults but is even higher in immunocompromised individuals and in those with neurologic diseases.^12^ Although its pathobiology has not been precisely determined, the most common etiology of SD is *Malassezia* yeast infection. Other potential causative factors include compromise of epidermal barrier integrity or skin microbiota composition, androgen or sebaceous activity, host immune response, and environmental changes.^13^ Currently, SD is mainly treated with anti‐inflammatory medication and topical corticosteroid agents.

Since osteoporosis and SD share a similar pathobiology, e.g. inflammation and hormonal imbalance, the aim of this study was to use the Taiwan National Health Insurance Research Database (NHIRD) to investigate whether the existence of SD increases osteoporosis risk.

## 2 METHODS

### 2.1 Data sources

The Taiwan National Health Insurance (NHI) program implemented in March 1995 covers >99% of the 23.74 million residents of Taiwan. The NHIRD published by the National Health Research Institute is an encrypted secondary database containing all records for the NHI program. The NHIRD is a large‐sample sized database of real‐world evidence made available for use in medical research. This study was performed using the Longitudinal Health Insurance Databases (LHID) 2010, which contains data for 1 000 000 beneficiaries randomly sampled from the original NHIRD. All diseases were coded based on the International Classification of Diseases, Ninth Revision, Clinical Modification (ICD‐9‐CM).

### 2.2 Study population

This study recruited 7831 patients aged 18–50 years with SD defined as a record of ICD‐9‐CM codes 706.3 and 690.1 entered by a dermatologist during 1996–2010 in two or more consecutive ambulatory visits or in one or more inpatient visits. The index date was the date of the first SD diagnosis. Propensity score matching was used to match the SD group with a control group of 31 324 patients without SD by age, gender, Charlson Comorbidity Index (CCI), and index date at a ratio of 1:4.

### 2.3 Main outcome

Both the SD group and the non‐SD group (controls) were followed up until the date of the first osteoporosis diagnosis or until December 31, 2010. An osteoporosis diagnosis was defined as an ICD‐9‐CM code 733.0 entered by an orthopedic surgeon in two or more consecutive ambulatory visits or in one or more inpatient visits and at least one record of a bone mineral density examination.^14^, ^15^, ^16^, ^17^, ^18^, ^19^, ^20^, ^21^

### 2.4 Comorbidities

Baseline comorbidities identified as potential confounders in this study were diabetes mellitus (ICD‐9‐CM code 250), hyperlipidemia (ICD‐9‐CM code 272), hypertension (ICD‐9‐CM codes 401–405), chronic liver disease (ICD‐9‐CM codes 456, 571, and 572), hyperthyroidism (ICD‐9‐CM code 242), chronic kidney disease (ICD‐9‐CM codes 582, 583, 585, 586, and 588), chronic pulmonary disease (ICD‐9‐CM codes 490–496), depression (ICD‐9‐CM codes 296.2, 296.3, 300.4, and 311), stroke (ICD‐9‐CM codes 430–438), epilepsy (ICD‐9‐CM code 345), dementia (ICD‐9‐CM codes 290, 294.1, 331.0, and 331.2), and psoriasis (ICD‐9‐CM code 696.0, 696.1, and 696.8). The severity of comorbidities was classified into four levels according to the CCI: 0, 1–2, 3–4, and >5.

### 2.5 Statistical analysis

Chi‐square test and Student's *t*‐test were used as appropriate in comparisons of categorical and continuous variables of demographic characteristics between the SD and non‐SD groups. In each group, overall incidence rates specific to gender and age were estimated per 1000 person‐years. The cumulative incidence of osteoporosis was determined in each group using the Kaplan–Meier method and compared between groups using the log‐rank test. Cox proportional hazard regression models were used to compare hazard ratio (HRs) and 95% confidence intervals (CIs) for osteoporosis between the two groups with adjustments for age, gender, CCI, and relevant comorbidities (diabetes mellitus, hyperlipidemia, hypertension, chronic liver disease, hyperthyroidism, chronic kidney disease, chronic pulmonary disease, depression, stroke, epilepsy, dementia, and psoriasis) in the multivariable model. A *P* value <0.05 was considered statistically significant. Statistical Analysis Software 9.4 (SAS Institute, Cary, NC, USA) was used to process all data analyses.

## 3 RESULTS

The 39 155 patients enrolled in this study included 7831 patients with SD (SD group) and a control group of 31 324 without SD (non‐SD group). In each group, the majority (53.74%) of patients was female (Table 1). Compared to the non‐SD group, the SD group had a higher prevalence of diabetes mellitus, hyperlipidemia, hypertension, chronic liver disease, hyperthyroidism, depression, stroke, dementia, and psoriasis.

**TABLE 1:** Demographic data between patients with seborrheic dermatitis and controls

Characteristics	With seborrheic dermatitis (*n* = 7831)	Controls (*n* = 31 324)	*P*
Mean age at enrolled (SD), years	33.1 (9.0)	33.3 (9.1)	0.1339
Age subgroup, *n* (%)			
18–29	3334 (42.57)	13 336 (42.57)	1.000
30–39	2327 (29.72)	9308 (29.72)	
40–49	2170 (27.71)	8680 (27.71)	
Gender, *n* (%)			
Males	3623 (46.26)	14 492 (46.26)	1.000
Females	4208 (53.74)	16 832 (53.74)	
Charlson Comorbidity Index, *n* (%)			
0	2307 (29.46)	9228 (29.46)	1.000
1–2	3725 (47.57)	14 900 (47.57)	
3–4	1292 (16.50)	5168 (16.50)	
≥5	507 (6.47)	2028 (6.47)	
Comorbidity, *n* (%)			
Diabetes mellitus	759 (9.69)	2402 (7.67)	<0.001
Hyperlipidemia	1628 (20.79)	4525 (14.45)	<0.001
Hypertension	1110 (14.17)	2991 (9.55)	<0.001
Hyperthyroidism	556 (7.10)	1529 (4.88)	<0.001
Chronic liver disease	2367 (30.23)	8175 (26.10)	<0.001
Chronic kidney disease	445 (5.68)	1738 (5.55)	0.644
Chronic pulmonary disease	2559 (32.68)	10 496 (33.51)	0.163
Depression	960 (12.26)	2543 (8.12)	<0.001
Stroke	165 (2.11)	472 (1.51)	<0.001
Epilepsy	133 (1.70)	523 (1.67)	0.859
Dementia	41 (0.52)	78 (0.25)	<0.001
Psoriasis	670 (8.56)	299 (0.95)	<0.001

Abbreviation: SD, standard deviation.

The incidence of osteoporosis was significantly higher in the SD group (0.98%, *n* = 77) compared to the non‐SD group (0.66%, *n* = 206) (Table 2). Additionally, the SD group tended to develop osteoporosis more rapidly (2.2 years after enrolment) compared to the control group (8.9 years after enrolment).

**TABLE 2:** Characteristic of osteoporosis events between patients with seborrheic dermatitis and controls

Characteristics	With seborrheic dermatitis (*n* = 7831)	Controls (*n* = 31 324)	*P*
Osteoporosis event, *n* (%)	77 (0.98)	206 (0.66)	0.002
Period of developing osteoporosis median (IQR), years	2.2 (1.1–5.1)	8.9 (5.5–12.2)	<0.001

Abbreviation: IQR, interquartile range.

Table 3 shows that, during the follow‐up period, the SD group had a 5.95‐fold higher osteoporosis risk compared to the non‐SD group (2.42 vs 0.44 per 1000 person‐years, respectively) after adjustment for age, gender, CCI, and comorbidities. Stratified analysis also revealed a higher osteoporosis risk in the SD group compared to the non‐SD group. In gender‐specific analyses of SD patients, the incidence of osteoporosis was higher in females compared to males (3.69 vs 1.04 per 1000 person‐years, respectively); in addition, osteoporosis risk in SD patients was higher in females compared to males (adjusted HR = 7.36, 95% CI = 5.15–10.52 for females; adjusted HR = 3.80, 95% CI = 1.95–7.38 for males). In age‐specific analysis, the incidence of osteoporosis substantially and consistently increased with age. However, the impact of SD on osteoporosis risk was higher in younger‐aged patients (adjusted HR = 8.05, 95% CI = 4.28–15.12) than in older‐aged patients (adjusted HR = 6.16, 95% CI = 4.31–8.81).

**TABLE 3:** Comparison of osteoporosis events between patients with seborrheic dermatitis and controls

Variables	With seborrheic dermatitis			Controls			Crude HR^a^ (95% CI)	Adjusted HR^a^ (95% CI)	*P*
	Osteoporosis events	PYs	Rate	Osteoporosis events	PYs	Rate			
Overall	77	31 874.07	2.42	206	468 494.47	0.44	8.05 (5.93–10.92)	5.95 (4.35–8.13)	<0.001
Gender									
Males	16	15 325.62	1.04	55	216 992.88	0.25	6.12 (3.24–11.54)	3.80 (1.95–7.38)	<0.001
Females	61	16 548.45	3.69	151	251 501.59	0.60	9.15 (6.43–13.01)	7.36 (5.15–10.52)	<0.001
Age subgroup									
18–29	8	13 286.19	0.60	26	19 9854.95	0.13	6.78 (3.02–15.23)	6.03 (2.68–13.58)	<0.001
30–39	15	9258.64	1.62	35	139 398.47	0.25	9.39 (5.02–17.54)	8.05 (4.28–15.12)	<0.001
40–49	54	9329.24	5.79	145	129 241.04	1.12	7.31 (5.17–10.34)	6.16 (4.31–8.81)	<0.001

Abbreviations: 95% CI, 95% confidence interval; HR, hazard ratio; PY, person‐year; Rate, incidence rate in per 1000 person‐years.

^a^ Model adjusted for age, gender, CCI and relevant comorbidities.

**FIGURE 1:**
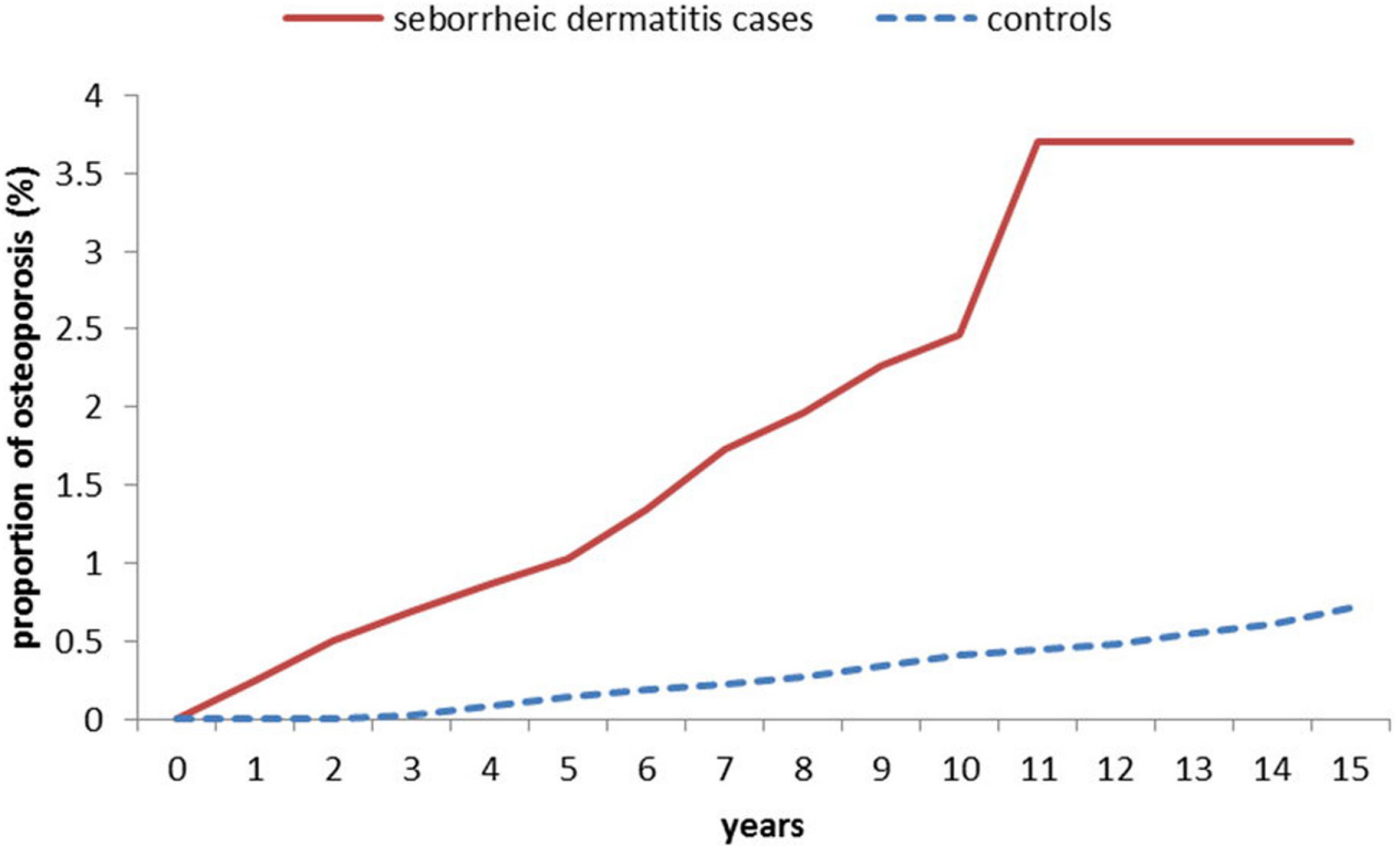
Comparison of Kaplan–Meier curves for osteoporosis risk between patients with seborrheic dermatitis and controls during the 15‐year follow‐up period

Figure 1 shows that the Kaplan–Meier method with log‐rank test further revealed a significantly higher cumulative incidence rate of osteoporosis in the SD group compared to the non‐SD patients (*P* < 0.001).

Table 4 shows that, in patients diagnosed with SD, the risk factors for osteoporosis included advanced age, female, high CCI, hyperlipidemia, hyperthyroidism, and epilepsy.

**TABLE 4:** Significant predictors of osteoporosis after seborrheic dermatitis diagnosis

Variables	Adjusted HR^a^	95% CI	*P*
Age	2.56	(1.78–3.70)	<0.001
Sex	4.44	(2.53–7.79)	<0.001
Charlson Comorbidity Index	1.37	(1.05–1.79)	0.019
Hyperlipidemia	2.13	(1.28–3.54)	0.004
Hyperthyroidism	2.02	(1.16–3.50)	0.012
Epilepsy	4.09	(1.76–9.50)	0.001

Abbreviations: 95% CI, 95% confidence interval; HR, hazard ratio.

a The adjusted HR and 95% CI were estimated by a stepwise Cox proportional hazards regression method; model adjusted for age, gender, CCI and relevant comorbidities.

## 4 DISCUSSION

This study was the first large retrospective cohort study to demonstrate an association between SD and osteoporosis: 0.98% of SD patients aged 18–50 years had osteoporosis. This SD group has a 5.95‐fold higher risk of osteoporosis than the control cohort after adjustment for comorbidity. Among the SD group, osteoporosis was more common in females than in males. Additionally, the effect of SD on osteoporosis was more prominent in younger patients than older patients. Hyperlipidemia, hyperthyroidism, and epilepsy synergistically increased the incidence of osteoporosis in the SD group.

The mechanism of the increased osteoporosis risk in SD patients is likely multifactorial. First, *Malassezia* yeasts are already known to be a major causative factor in SD. In *Malassezia* species, lipase secretion hydrolyses free fatty acids, which induces inflammation by generating large quantities of oleic acids and arachidonic acids.^22^ By inducing assembly of inflammasomes and activation of pattern recognition receptors, immune system dysregulation causes keratinocyte differentiation and proliferation.^23^, ^24^ Studies of SD patients also show that high androgen activity causes sebaceous gland activity and lipid composition, which then induces *Malassezia* proliferation and a continual cycle of inflammation.^13^, ^25^ Hence, the receptor activator of nuclear factor κB ligand (RANKL), tumor necrosis‐α, and interleukin‐6 are not only inflammatory cytokines, but also pro‐osteoclastogenic factors that promote osteoclast activation and differentiation.^26^ In a mouse model of skin inflammation, *KCASP1Tg* mice exhibited osteoporosis and increased expression of inflammatory cytokines in the femur.^27^ Hence, SD increases osteoporosis risk by generating an inflammatory response.

Second, since both SD and psoriasis are diseases of chronic inflammation, SD patients and psoriasis patients share common clinical characteristics, and co‐presence of SD and psoriasis is occasionally reported.^28^, ^29^ By examining employees in a voluntary company database in Germany, Zander et al. reported that approximately 2.7% of SD patients had psoriasis, suggesting that SD patients had a higher prevalence of psoriasis (odds ratio [OR] = 1.2, 95% CI = 1.0–1.5).^29^ In a cross‐sectional study through analyzing the Clalit Health Services database in Israel, SD patients were more likely to have psoriasis (1.6% vs 0.8%).^28^ In our study, SD patients were also prone to develop psoriasis. Psoriasis is a is not only a chronic multisystem inflammatory disease but is also related to vitamin D deficiency. Keller et al. have shown that osteoporotic patients had a higher prevalence of psoriasis (OR = 1.65, 95% CI = 1.42–1.94).^18^ Additionally, psoriasis was reported to be related to male osteoporotic patients (OR = 1.86, 95% CI = 1.44–2.39) in a case–control study analyzing a database in Israel.^30^ Attia et al. reported that psoriasis patients with or without arthritis were prone to have lower bone mineral density than healthy people.^31^ Antonio et al. demonstrated that patients with long‐term psoriasis tended to develop decreased bone mineral density relative to healthy individuals.^32^ In the meta‐analysis by Su et al., psoriatic patients were likely to have fractures relative to nonpsoriatic people (adjusted OR = 1.09, 95% CI = 1.06–1.12).^33^ Also, in a cross‐sectional study of Arias‐Santiago et al., patients with psoriatic disease had lower bone mineral density than healthy people.^34^ By analyzing the Korean National Health Insurance Service – Health Screening Cohort, Choi et al. reported that psoriasis increased the risk of osteoporosis (OR = 1.21, 95% CI = 1.16–1.27).^35^ Patients with extensive and chronic psoriasis are known to have increased risks of osteopenia and osteoporosis.^36^ Studies indicate that SD is also a predictor of metabolic syndrome,^37^ which is characterized by a cluster of disorders, including abdominal obesity, hypertension, hyperlipidemia, and insulin resistance. Since insulin resistance and bone metabolism share a similar pathophysiology, abnormal signaling of insulin could cause dysregulation of osteoblast activity and osteoclast differentiation, leading to bone damage and osteoporosis.^9^, ^10^ Third, SD is readily aggravated by stress. A cross‐sectional study of a Chinese population of SD patients reported that nearly half of patients had severe emotional disorders,^38^ including anxiety, depression, and obsessive–compulsive disorder.^39^ In osteoporosis patients, depression can contribute to bone loss and osteoporotic fractures by activating the hypothalamo–pituitary–adrenal (HPA) axis and sympathetic system.^40^, ^41^ The SD patients in this study had a high prevalence of depression, hypertension, hyperlipidemia, and diabetes mellitus. Seborrheic dermatitis may contribute to osteoporosis risk by increasing the risks of metabolic syndrome, psoriasis, and depression.^42^ However, these complex associations need further clarification.

In our study, the influence of SD on osteoporosis risk was higher in patients aged 30–39 years compared to other age groups. In all SD patients, the osteoporosis incidence rate increased with age. Compared to younger patients, however, older patients are more likely to have multiple comorbidities that contribute to osteoporosis. Thus, the role of SD in accelerating bone loss may not significantly increase with age. These data indicate that the pathobiological role of SD in osteoporosis may differ by age. Patients in puberty or in younger age groups are in a vulnerable phase of skeletal bone mineralization. In younger age groups, a high rate of vitamin D turnover results in a high prevalence of hypovitaminosis D.^43^ Abnormally high vitamin D turnover also decreases osteoblast function, which causes deficient bone acquisition.^44^

Moreover, the osteoporosis incidence in SD patients is reportedly increased in the presence of hyperlipidemia, hyperthyroidism, and epilepsy. The biologically active thyroid hormone 3,5,3′‐l‐triiodothyronine regulates the rates of bone maturation and mineralization.^45^ The accelerated bone turnover rate in hyperthyroidism patients increases bone resorption and mineralized bone lost.^46^, ^47^, ^48^ Cholesterol and its metabolites also affect bone homeostasis by modulating the activation and differentiation of osteoblasts and osteoclasts.^49^ Oxidized lipid accumulation in bone tissues attenuates osteogenic differentiation and parathyroid hormone resistance, both of which promote osteoporosis.^50^, ^51^ Serum lipid levels negatively correlate with bone mineral density.^51^ Notably, people with epilepsy are often deficient in vitamin D, which decreases bone mineral density and increases fracture risk. In epilepsy patients, the osteoporosis rate tends to be highest in young age groups and in patients treated with multiple antiepileptic drugs,^52^, ^53^ therefore concomitant diseases in patients with SD may be predictive factors for osteoporosis, which leads to increased fracture.^46^, ^48^, ^54^, ^55^, ^56^

The biggest strength of our study is that it is the first population‐based study with a large sample size to investigate the interplay between SD and osteoporosis risk. However, a limitation is that diagnoses of SD and osteoporosis were both defined by ICD‐9‐CM codes from claims records. Hence, the numbers of patients with SD and patients with osteoporosis may have been underestimated because patients with untreated SD are also at risk of undiagnosed osteoporosis. If the SD patients have higher consciousness of medical problems than non‐SD patients, they are keen to receive osteoporosis examination and have more chance to be diagnosed as osteoporosis. Yet, Taiwanese national health insurance offers far lower payments to boast universal health coverage and easy access to any medical institutions. Although we could not completely exclude the interference of the increased exposure of SD patients to the medical community, most Taiwanese are indeed quick to seek medical attention when suffering from any discomfort based on the convenience and low cost of the health system. To minimize the bias, we also utilized Cox proportional hazard regression models to compare the hazard ratio and 95% confidence intervals for osteoporosis between the two groups with adjustment for age, gender, CCI, and relevant comorbidities in the multivariable model. These limitations are common in studies that have used electronic health insurance databases in other countries. Since the dataset did not include SD severity, the association between SD severity and outcome could not be determined. However, the LHID has already been used for various scientific studies of osteoporosis risk factors^57^, ^58^, ^59^; our study provides additional scientific information. A final limitation is that the database lacked detailed data for osteoporosis risk factors that may have influenced the results, including relevant genetic factors, family history, sun exposure, diet/exercise habits, tobacco and alcohol consumption, and body mass index.^58^

In conclusion, this study is the first cohort study to demonstrate that SD patients have an increased risk of osteoporosis, especially in the presence of hyperlipidemia, hyperthyroidism, and epilepsy. Notably, the influence of SD on osteoporosis risk was largest in young age groups. Since the influence of bone mass and overall bone health in SD patients with concomitant diseases is assumedly largest at an early age, lifestyle and diet recommendations as well as regular bone metabolism/mineral density examinations are warranted in routine treatment of SD.

## CONFLICT OF INTEREST

None declared.

## ETHICS STATEMENT

All insurance reimbursement claims data used in this study were collected from the Taiwan NHIRD. The study was assessed and approved by the Institutional Review Board of Kaohsiung Medical University Hospital (KMUHIRB‐EXEMPT‐[I] 20150040) in accordance with Declaration of Helsinki principles. Based on the regulations of the Institutional Review Board, the informed consent requirement was waived.
